# An Outbreak of Carbapenem-Resistant *Klebsiella pneumoniae* of K57 Capsular Serotype in an Emergency Intensive Care Unit of a Teaching Hospital in China

**DOI:** 10.3389/fpubh.2021.724212

**Published:** 2021-09-01

**Authors:** Chunhong Shao, Yan Jin, Wei Wang, Meijie Jiang, Shuping Zhao

**Affiliations:** ^1^Clinical Laboratory of Shandong Provincial Hospital Affiliated to Shandong First Medical University, Jinan, China; ^2^Intensive Care Department of Taian City Central Hospital, Tai'an, China; ^3^Clinical Laboratory of Taian City Central Hospital, Tai'an, China

**Keywords:** *Klebsiella pneumoniae*, K57, hypervirulent, carbapenem-resistant, outbreak, China

## Abstract

The emergence of carbapenem-resistant hypervirulent *K. pneumoniae* (CR-hvKP) strains has increased the threat posed by *K. pneumoniae*. Here, we described an outbreak of 32 CR-hvKP isolates from the emergency intensive care unit (EICU) of a teaching hospital in China. Thirty-two CRKp isolates were collected from six patients and their surrounding environment in EICU. Antimicrobial susceptibility testing was performed using VITEK 2 compact system, E-test or the broth microdilution method. All isolates were serotyped, antimicrobial resistance genes and virulence-associated genes were screened using PCR. Multilocus sequence typing (MLST) and pulse-field gel electrophoresis (PFGE) were employed to characterize the genetic relationships among the CPKP isolates. The virulence capability of 11 CRKp isolates from six patients was evaluated through *Galleria mellonella* larva infection assay. PFGE showed that all 32 isolates belonged to one cluster, and MLST revealed that belonged to ST11. All isolates exhibited high resistance to β-lactam antibiotics, quinolones, and aminoglycosides. They were susceptible to ceftazidime/averbatan, tigecycline, and colistin. All 32 isolates harbored *bla*_KPC−2_, *bla*_SHV−11_, *bla*_TEM−1_, *rmtB*, and *qnrD*. The serotype of all 32 isolates was K57. All 32 isolates contained 6 virulence genes, namely, *fimH, iucB, mrkD, rmpA, uge*, and *wabG*. Infection assays demonstrated high mortality in the *Galleria mellonella* model. Following measures implemented by the hospital, the outbreak was controlled. The mortality rate was 50.0%. The epidemiology of CR-hvKP should be monitored closely to detect early indications of this emerging public health threat.

## Introduction

*Klebsiella pneumoniae* is a common causative pathogen of various nosocomial infections, including pneumonia, urinary tract infection, abdominal infection, and bacteremia. *K. pneumoniae* is now recognized as an urgent threat to human health because of the emergence of multidrug-resistant strains associated with hospital outbreaks and hypervirulent strains associated with severe community-acquired infections (1). Surveillance of antibiotic resistance by CHINET in China showed that 4.9 and 4.8% of *K. pneumoniae* strains were resistant to imipenem and meropenem, respectively, in 2009, compared with 25.3 and 26.8%, respectively, in 2019 (http://chinets.com/Data/GermYear). Infections caused by carbapenem-resistant *K. pneumoniae* (CRKp) have few treatment options and are associated with mortality rates upwards of 50% (2). Several mechanisms are responsible for the carbapenem-resistance of *K. pneumoniae*, among which, carbapenemase production remains the most clinically relevant (3).

Over the past two decades, the newly emerged hypervirulent *K. pneumoniae* (hvKP) strain has caused great concern globally. HvKp is an evolving pathotype that is more virulent than classical *K. pneumoniae* (cKP). HvKP usually infects healthy individuals from the community. Infections are more common in the Asian but are occurring globally (4). Unlike cKP, hvKP is rarely resistant to commonly used antimicrobial agents, except for an intrinsic resistance to ampicillin (5). The distinct virulence determinants of hvKp include iron acquisition, increased capsule production, K1 and K2 capsule types, and the colibactin toxin (6). The ability to acquire iron is essential for bacterial growth and the progression of infection. *iucABCD*, which encodes for aerobactin and its cognate receptor, is more prevalent in hvKP than cKP (7). Additionally, hvKp strains demonstrate hypermucoviscosity, a phenotypic description of hvKp in laboratory conditions that has become a distinguishing feature of many hypervirulent isolates (6).

Along with the global dissemination of mobile genetic elements conferring antibiotic resistance, antibiotic-resistant hvKP isolates, especially carbapenem-resistant hvKP (CR-hvKP), have attracted increasingly more attention and have been increasingly reported worldwide (5). In China, several outbreaks of CR-hvKP have been reported (8–10). *bla*_KPC−2_ is the predominant carbapenemase in both CRKp and CR-hvKp (11, 12). ST11 is the major sequence type of CR-hvKp from Asia, especially China (13). Here, we have described an outbreak of CR-hvKP with 32 isolates from the emergency intensive care unit (EICU) of Taian City Central Hospital, Shandong, China. We sought to investigate the molecular and epidemiological features of these isolates during the outbreak, especially the characteristics of antimicrobial resistance determinants and virulence factors.

## Materials and Methods

### Patients and CRKp Isolates From EICU

From January 29, 2019 to March 11, 2019, six patients with CRKp infection were found in EICU. A total of 11 CRKps were isolated from their blood, sputum, and bronchoalveolar lavage fluid (BALF). Patient information including age, gender, length of EICU stay, diagnosis, treatment, and outcomes were obtained from electronic medical records. The methods used in this study were approved by the Ethics Committee of Taian City Central Hospital and were carried out in accordance with the approved guidelines. On February 10 and February 20, 2019, the hospital environment of six patients was sampled and 21 CRKp isolates were obtained from the patients' environment in the hospital, including bed sheets, pillows, sphygmomanometers, ventilators, tables, mice, keyboards, and the hands of medical staff. The 32 isolates were identified as *K. pneumoniae* using Vitek-MS system (BioMérieux, France). Phenotypic detection of carbapenemases was performed using carbapenem inactivation method (CIM) and EDTA-modified CIM (eCIM) test according to the approved standard of the Clinical and Laboratory Standards Institute 2020 guidelines (14).

### Determination of Hypermucoviscosity Phenotype

The hypermucoviscosity phenotype was determined by the “string test.” Samples were cultured on agar plates overnight at 37°C. Then, a colony from the plate was stretched with a bacteriology loop. If a viscous string over 5 mm in length forms, it is considered to be a positive result.

### Antibiotic Susceptibility Assay

Antimicrobial susceptibility testing was performed using VITEK 2 compact system (BioMérieux, France). The minimum inhibitory concentrations (MICs) of imipenem, meropenem, and ertapenem were determined through an E-test (BioMérieux, France). The MICs of tigecycline and colistin were determined using the broth microdilution method (Bio-kont, China). The antimicrobial susceptibility of ceftazidime/averbatan was determined using Kirby-Bauer test (Oxide, America). *Escherichia coli* ATCC25922 and *K. pneumoniae* ATCC700603 served as the quality controls. All antibiotics were administered according to the approved standard of the 2020 European Committee on Antimicrobial Susceptibility Testing breakpoint (www.eucast.org/clinical-breakpoint).

### PCR and DNA Sequence Analysis of Drug Resistance Genes, Serotype, and Virulence Genes

As described previously, a variety of antimicrobial resistance genes were screened using PCR and DNA sequencing (15). These genes included carbapenem resistance genes (*bla*_KPC_*, bla*_SME_*, bla*_IMI_*, bla*_NMC_*, bla*_GES_*, bla*_IMP_*, bla*_VIM_*, bla*_GIM_*, bla*_SIM_*, bla*_SPM_*, bla*_NDM_, and *bla*_OXA−48like_), other β-lactamase genes (*bla*_CTX−M_*, bla*_TEM_*, bla*_SHV_, *bla*_MOX_*, bla*_FOX_*, bla*_DHA_*, bla*_CIT_*, and bla*_EBC_), plasmid-mediated quinolone resistance genes [*qnrA, qnrB, qnrC, qnrD, qnrS, qepA, and aac* *(**6**)-Ib-cr*], and 16S rRNA methyase gene *(rmtA, rmtB, rmtC, rmtD, npmA*, and *armA*). The positive PCR products were sequenced by Beijing Genomics Institute Technology Co. Ltd. Sequence alignments were completed by running BLAST at NCBI website (http://blast.ncbi.nlm.nih.gov/Blast.cgi). Subsequently, the isolates were serotyped for K1, K2, K5, K20, K54, and K57 serotypes, and twelve virulence-associated genes, including *rmpA, aerobactin, wcaG, ybtA, iucB, iroNB, ureA, uge, kfuBC, fim, wabG*, and *allS*, were screened using PCR, as previously described (15).

### Multilocus Sequence Typing

MLST of *K. pneumoniae* was performed according to protocols available on the MLST Pasteur website (http://www.pasteur.fr/recherche/genopole/PF8/mlst/Kpneumoniae.html). Seven conserved housekeeping genes (*gapA, infB, mdh, pgi, phoE, rpoB*, and *tonB*) were amplified, sequenced, and compared with those in the MLST databases.

### Pulse-Field Gel Electrophoresis

An overnight bacterial culture was suspended in cell suspension buffer [100 mM EDTA, 100 mM Tris-HCl (pH 8.0)] and adjusted to an optical density of 4.0 at the wave length of 600 nm. The suspension was mixed with equal volume of 2% solution of low-melting agarose in Tris-EDTA [TE: 1 mM EDTA, 10 mM Tris-HCl (pH 8.0)]. After cooling, the agarose sections were incubated for 4 h at 54°C in cell lysis buffer [50 mM Tris-HCl, 50 mM EDTA (pH 8.0), 0.01 g/mL N-lauroyl-sarcosine, sodium salt, 0.1 mg/mL proteinase K]. They were then washed thoroughly with TE buffer and digested overnight with XbaI restriction endonuclease (Takara Bio, Inc., Otsu, Japan). Genomic DNA separation was performed in 0.5 X Tris/borate/EDTA (TBE) buffer in a PFGE system (CHEF Mapper; Bio-Rad Laboratories, Inc., Hercules, CA, USA) at 14°C, using a voltage of 6 V/cm, a switch angle of 120° and a switch ramp of 6–36 s for 19 h. The *Salmonella enterica* serotype Braenderup H9812 was used as a marker for PFGE.

### Galleria Mellonella Larva Infection Assay

The virulence capability of 11 CRKp isolates from six patients was evaluated through a commonly used *in vivo* infection model, the wax moth *G. mellonella* (8). In the present study, each infection group included 10 larvae. Larvae were injected with 10 μL of bacterial suspension at a concentration of 10^6^ CFU per larva. As a control group, 10 μL PBS was injected in parallel. All larvae were placed in petri-dishes and kept at 37°C in the dark. At 24, 48, 72, and 96 h after injection, the number of dead larvae was recorded with notes on any melanization and lack of motility. All experiments were performed with biological triplicates and results were not considered if two or more larvae in any of the control groups died.

## Results

### Outbreak Description

**Figure 1:**
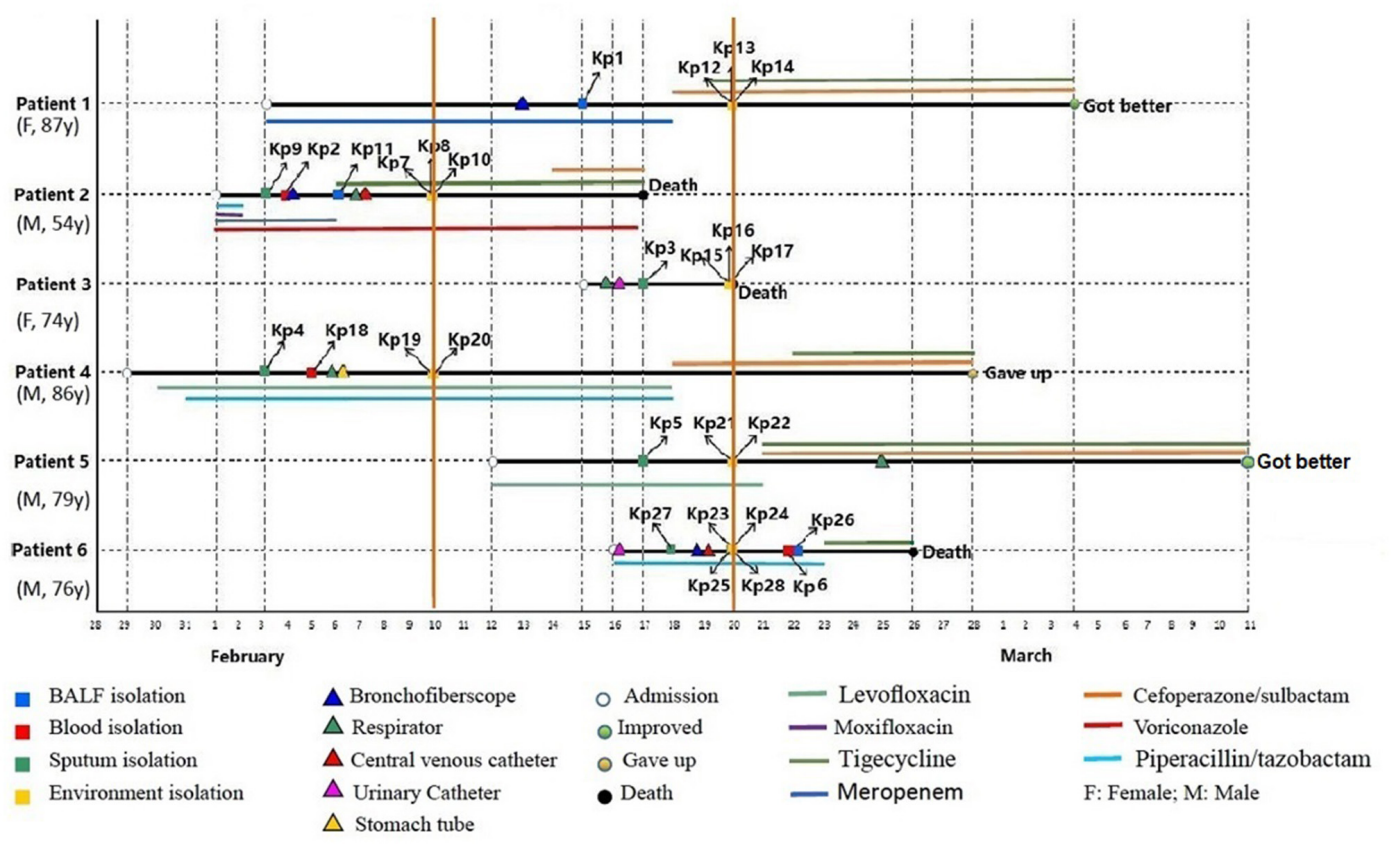
Epidemiology of the CR-hvKP outbreak cases. Each horizontal line represents the timeline of one patient, with the abscissa axis showing the relevant dates. The isolates from different specimens and the traumatic treatment of these patients are represented by “□” and “Δ,” respectively. The outcome of the patients was represented by “O,” and the antibiotics used were represented by different color lines, and the length of the color lines represented the length of the antibiotic use.

From January 29 to March 11, 2019, six patients admitted to EICU were included in this analysis. The age of patients ranged from 54 to 87 years and the patients were admitted to EICU due to Chronic obstructive pulmonary disease (COPD), severe pneumonia, cerebral infarction, and septic shock. They all showed the typical symptoms of pulmonary edema, excessive sputum, pleural effusion, and shortness of breath (Table 1). All six patients received traumatic and/or antibiotic treatment during their hospitalization in the EICU (Figure 1). The antibiotics used mainly included carbapenems, cefoperazone/sulbactam, piperacillin/tazobactam, quinolones, and tigecycline. In addition, patient 2 was treated with voriconazole.

**Table 1:** Clinical characteristics of six patients with CRKp infection in EICU.

**No**.	**Gender**	**Age**	**Clinical diagnosis**	**CRKp derived specimens**	**Antibiotic therapy**	**Admission date**	**Discharge date**	**Traumatic treatment**	**Prognosis**
P1	F	86	Severe pneumonia	BALF	MEM, SCF,TGC	2019/2/3	2019/3/4	Bronchoscope	Got better
P2	M	66	Septic shock	Blood, Sputum, BALF	TZP, MFX, VRC, LEV, TGC, SCF	2019/2/1	2019/2/17	Respirator, Bronchoscope, PICC	Death
P3	F	85	COPD	Sputum	N	2019/2/15	2019/2/20	Respirator, Urinary catheter	Death
P4	M	81	Severe pneumonia	Blood, Sputum	TZP, LEV, SCF, TGC	2019/1/19	2019/2/28	Respirator, Gastric tube	Gave up
P5	M	77	COPD	Sputum	LEV, SCF, TGC	2019/2/12	2019/3/11	Respirator	Got better
P6	M	60	Cerebral infarction	Blood, Sputum, BALF	TZP, TGC	2019/2/16	2019/2/26	Respirator, Urinary catheter, PICC	Death

*F, female; M, man; N, no; COPD, Chronic obstructive pulmonary disease; BALF, Bronchoalveolar lavage fluid; MEM, meropenem; SCF, cefoperazone/sulbactam; TGC, tigecycline; TZP, piperacillin-tazobactam; MFX, moxifloxacin; VRC, voriconazole; LEV, levofloxacin; PICC, peripherally inserted central venous catheter*.

On February 3, 2019, the first two CRKp isolates were isolated from the sputum of patient 4 and patient 2, respectively. Patient 4 was the first patient to be admitted to EICU. Subsequently, CRKps were also isolated from blood samples or BALF of these two patients. So on February 10, the hospital environment of patient 2 and patient 4 was sampled and their rooms were thoroughly disinfected with glutaraldehyde. However, in the next 10 days, another 4 patients were continuously confirmed to have CPKp infection in the ECU. Among them, patient 1 was admitted to EICU as early as February 3, and the other three patients were admitted after February 10. Therefore, an overlap period between patient 1 and patient 2, patient 4 was observed. So on February 20, the hospital environment of patient 1, patient 3, patient 5, and patient 6 was sampled and their rooms were thoroughly disinfected with glutaraldehyde.

In order to control the outbreak, the staffs of EICU were trained and educated in environmental disinfection and carrying out hand hygiene in a timely and effective manner. Eventually, the epidemic situation was effectively controlled. Except for these 6 patients, no other infected patients have been found in EICU to date. Finally, two patients improved and was discharged, three patients died during treatment in hospital. The survival time of patient 3 was the shortest after CRKp isolation, which was only 3 days. One gave up treatment. The mortality rate was 50.0%.

### Drug Resistance and Resistance Genes of CRKp Isolates

The antimicrobial susceptibility profiles of the 32 CRKp isolates are listed in Supplementary Table 1. All isolates exhibited high resistance to aztreonam, cephalosporin, β-lactam/β-lactamase inhibitor combinations, carbapenems, quinolones, and aminoglycosides. Seven isolates (21.88%, 7/32) were resistant to trimethoprim-sulfamethoxazole. All isolates were susceptible to colistin, tigecycline, and ceftazidime/averbatan (100%). All strains were positive for MCIM test and negative for eCIM test.

For the resistance to β-lactams, all 32 isolates carried the *bla*_KPC−2_, *bla*_SHV−11_, and *bla*_TEM−1_ genes (Table 2). In addition, 27 isolates carried *bla*_CTX−14_ gene, and 3 isolates (Kp15, Kp16, and Kp17) from the hospital environment of patient 3 carried *bla*_CTX−14_ and *bla*_CTX−15_ simultaneously. For aminoglycoside resistance, all isolates carried *rmtB*, and three isolates also carried *armA*. In addition, all 32 isolates carried *qnrD*, which may have led to quinolone resistance.

**Table 2:** The drug-resistance genes and virulence genes of 32 CR-hvKP.

**Isolates**	**Drug-resistance genes**								**Virulence genes**					
	**β-lactamases**					**Aminoglycoside**		**Quinolone**	**Capsule**			**Fimbriae**		**Iron acquistion**
	***bla*_**KPC−2**_**	***bla*_**SHV−11**_**	***bla*_**TEM−1**_**	***bla*_**CTX−M14**_**	***bla*_**CTX−M15**_**	***rmtB***	***armA***	***qnrD***	***rmpA***	***wabG***	***uge***	***fimH***	***mrkD***	***iucB***
Kp1	⋆	⋆	⋆	⋆		⋆		⋆	⋆	⋆	⋆	⋆	⋆	⋆
Kp2	⋆	⋆	⋆	⋆		⋆		⋆	⋆	⋆	⋆	⋆	⋆	⋆
Kp3	⋆	⋆	⋆	⋆		⋆		⋆	⋆	⋆	⋆	⋆	⋆	⋆
Kp4	⋆	⋆	⋆	⋆		⋆		⋆	⋆	⋆	⋆	⋆	⋆	⋆
Kp5	⋆	⋆	⋆	⋆		⋆		⋆	⋆	⋆	⋆	⋆	⋆	⋆
Kp6	⋆	⋆	⋆	⋆		⋆		⋆	⋆	⋆	⋆	⋆	⋆	⋆
Kp7	⋆	⋆	⋆	⋆		⋆		⋆	⋆	⋆	⋆	⋆	⋆	⋆
Kp8	⋆	⋆	⋆	⋆		⋆		⋆	⋆	⋆	⋆	⋆	⋆	⋆
Kp9	⋆	⋆	⋆	⋆		⋆		⋆	⋆	⋆	⋆	⋆	⋆	⋆
Kp10	⋆	⋆	⋆	⋆		⋆		⋆	⋆	⋆	⋆	⋆	⋆	⋆
Kp11	⋆	⋆	⋆	⋆		⋆		⋆	⋆	⋆	⋆	⋆	⋆	⋆
Kp12	⋆	⋆	⋆	⋆		⋆	⋆	⋆	⋆	⋆	⋆	⋆	⋆	⋆
Kp13	⋆	⋆	⋆			⋆		⋆	⋆	⋆	⋆	⋆	⋆	⋆
Kp14	⋆	⋆	⋆	⋆		⋆		⋆	⋆	⋆	⋆	⋆	⋆	⋆
Kp15	⋆	⋆	⋆	⋆	⋆	⋆		⋆	⋆	⋆	⋆	⋆	⋆	⋆
Kp16	⋆	⋆	⋆	⋆	⋆	⋆		⋆	⋆	⋆	⋆	⋆	⋆	⋆
Kp17	⋆	⋆	⋆	⋆	⋆	⋆		⋆	⋆	⋆	⋆	⋆	⋆	⋆
Kp18	⋆	⋆	⋆	⋆		⋆		⋆	⋆	⋆	⋆	⋆	⋆	⋆
Kp19	⋆	⋆	⋆	⋆		⋆		⋆	⋆	⋆	⋆	⋆	⋆	⋆
Kp20	⋆	⋆	⋆	⋆		⋆	⋆	⋆	⋆	⋆	⋆	⋆	⋆	⋆
Kp21	⋆	⋆	⋆	⋆		⋆		⋆	⋆	⋆	⋆	⋆	⋆	⋆
Kp22	⋆	⋆	⋆	⋆		⋆		⋆	⋆	⋆	⋆	⋆	⋆	⋆
Kp23	⋆	⋆	⋆	⋆		⋆		⋆	⋆	⋆	⋆	⋆	⋆	⋆
Kp24	⋆	⋆	⋆	⋆		⋆	⋆	⋆	⋆	⋆	⋆	⋆	⋆	⋆
Kp25	⋆	⋆	⋆	⋆		⋆		⋆	⋆	⋆	⋆	⋆	⋆	⋆
Kp26	⋆	⋆	⋆	⋆		⋆		⋆	⋆	⋆	⋆	⋆	⋆	⋆
Kp27	⋆	⋆	⋆	⋆		⋆		⋆	⋆	⋆	⋆	⋆	⋆	⋆
Kp28	⋆	⋆	⋆	⋆		⋆		⋆	⋆	⋆	⋆	⋆	⋆	⋆
Kp29	⋆	⋆	⋆			⋆		⋆	⋆	⋆	⋆	⋆	⋆	⋆
Kp30	⋆	⋆	⋆	⋆		⋆		⋆	⋆	⋆	⋆	⋆	⋆	⋆
Kp31	⋆	⋆	⋆	⋆		⋆		⋆	⋆	⋆	⋆	⋆	⋆	⋆
Kp32	⋆	⋆	⋆	⋆		⋆		⋆	⋆	⋆	⋆	⋆	⋆	⋆

*⋆, positive; blank, negative*.

### Serotype and Virulence of CRKp Isolates

The string test of 32 isolates showed negative results. The serotype of all 32 isolates was K57. In the virulence gene analysis, all 32 isolates were found to contain 6 virulence genes, including *fimH, iucB, mrkD, rmpA, uge*, and *wabG* (Table 2).

**Figure 2:**
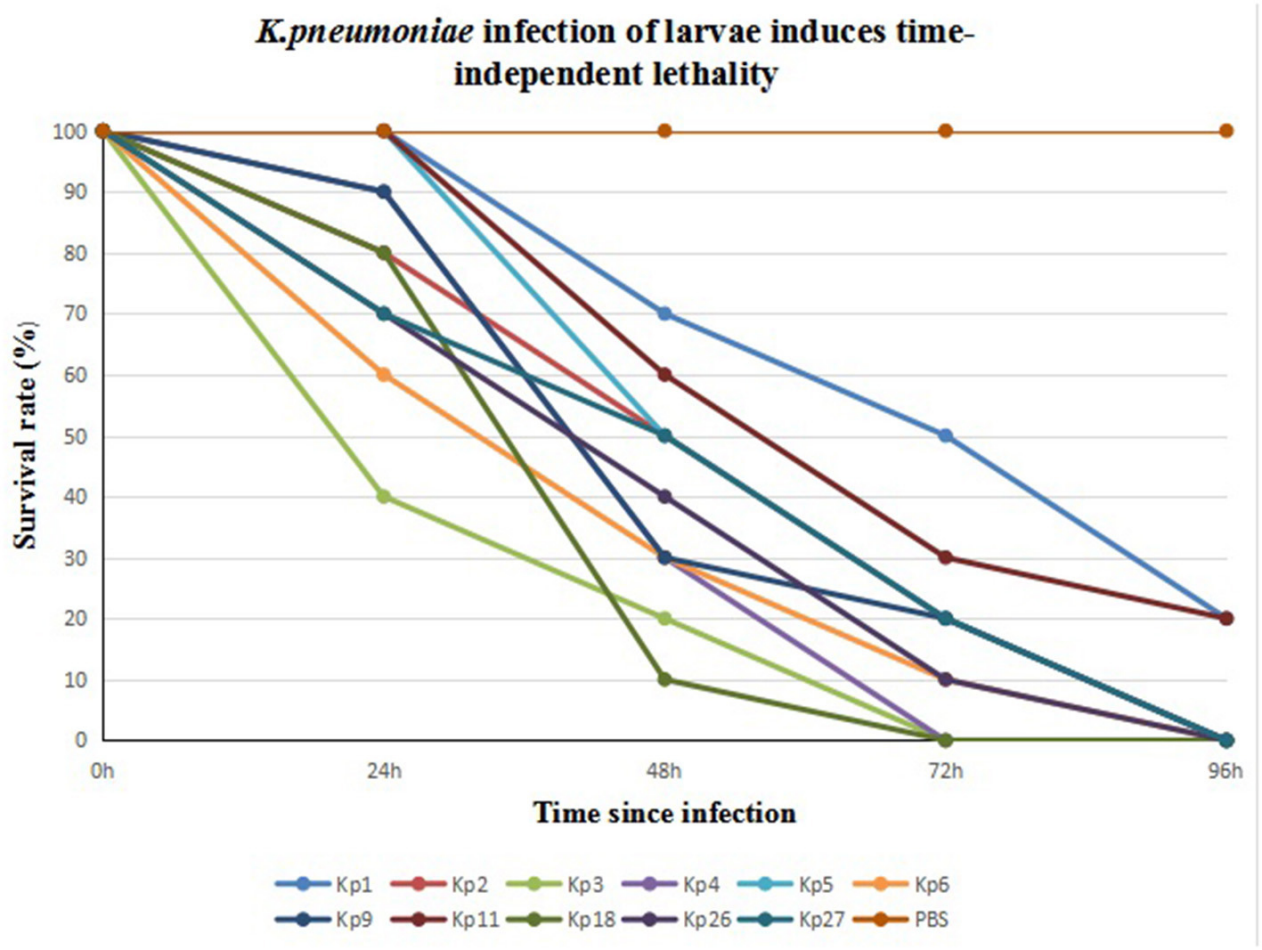
Eleven CR-hvKP isolates from six patients infection of *G. mellonella* larvae induces time-dependent lethality. The *G. mellonella* larvae injected with PBS were used as control.

We infected *G. mellonella* larvae with 11 CRKp isolates obtained from clinical specimens of the six patients. Kp1 and Kp11 from patient 1 were the least toxic, with 20% of larvae surviving after 96 h. All larvae infected by the other nine strains died within 96 h. Kp3 from patient 3 was the most toxic. The 24- and 48-h survival rates of larvae infected with Kp3 were 40 and 20% respectively, and all larvae died within 72 h. The results are shown in Figure 2.

### MLST and PFGE Analysis of CRKp Isolates

**Figure 3:**
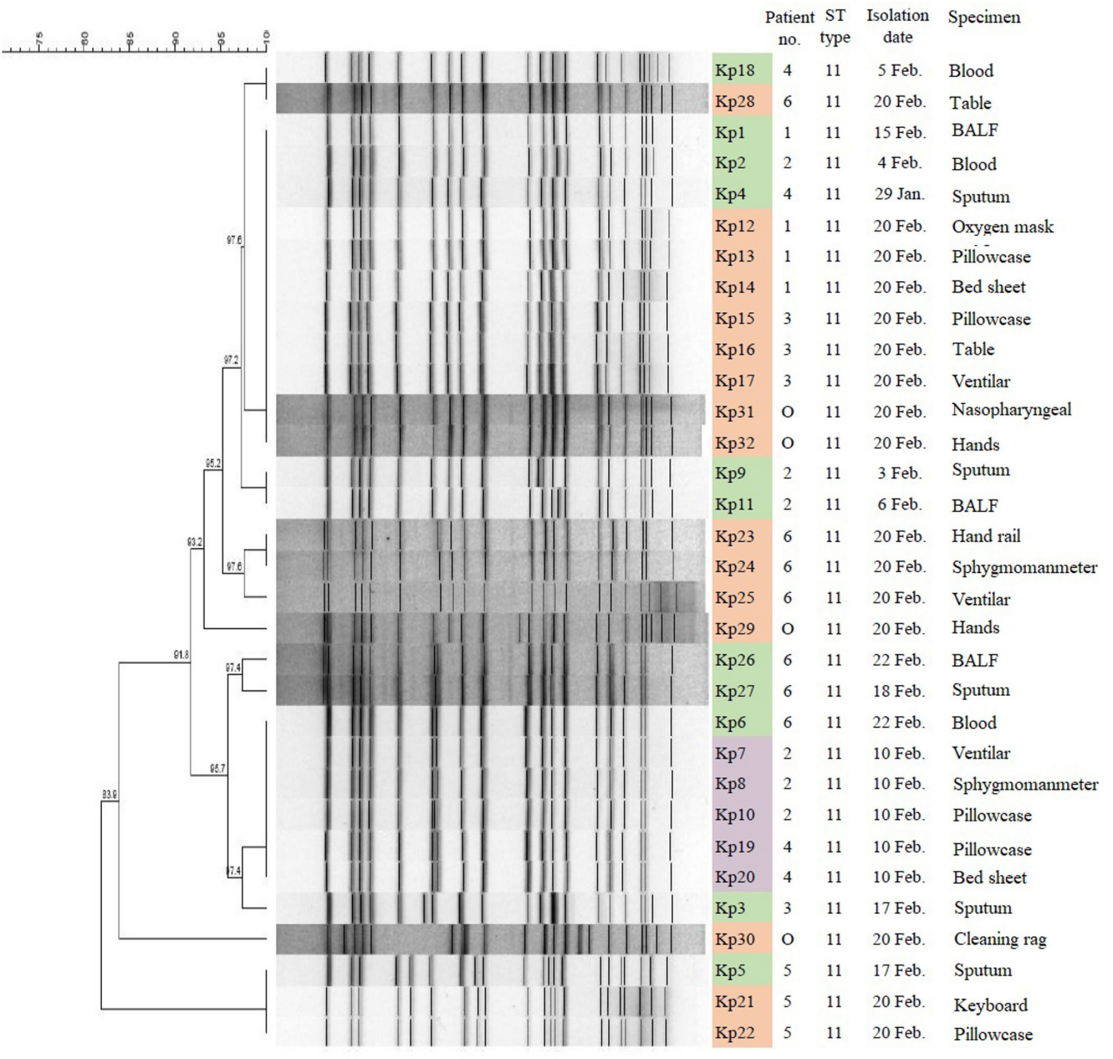
PFGE results and MLST typing for 32 CR-hvKP isolates. Green represents strains isolated from clinical specimens of six patients; purple represents strains isolated in hospital environment on February 10, 2019; and orange represents strains isolated in hospital environment on February 20, 2019. “O” represents strains isolated from hands of cleaner in EICU.

MLST analysis revealed that all 32 CRKp isolates were of the ST11 type, which is the most common type of CRKp found in China. The results of PFGE showed that the homology of 32 isolates was more than 80%, indicating that they were a cluster. With the exception of Kp5, Kp21, Kp22, and Kp30, the homology of other 28 isolates was higher than 91.8%. Furthermore, three isolates (Kp5, Kp21, and Kp22) from patient 5 and four isolates (Kp1, Kp12, Kp13, and Kp14) from patient 1 showed complete homology (Figure 3).

## Discussion

*K. pneumoniae* is widely recognized as a pathogen with a propensity for acquiring antibiotic resistance. It is capable of causing a range of hospital-acquired infections and community-acquired invasive infections (16). The appearance of CR-hvKP makes this bacterium more threatening. Here we described a nosocomial outbreak of CR-hvKP in the EICU of a teaching hospital. This outbreak involved six patients and lasted for 40 days. Thirty-two CR-hvKp were isolated from six patients and their surrounding environment. These six patients did not share one ward and did not share medical equipments. However, PFGE showed that all 32 CR-hvKp isolates belonged to one cluster. Hospital monitoring showed that this outbreak of CRKp might have been caused due to the contaminated hands of cleaners. The hospital took positive measures and the outbreak was controlled. Therefore, in order to prevent the spread and prevalence of multidrug-resistant bacteria, timely submission of specimens, and detection of multi-drug resistant bacteria are very important in addition to disinfection, isolation, and treatment (17). Previous and this study showed that *K. pneumoniae* could survive on hospital equipment and hands of medical staff that had undergone rigorous cleaning. It is suggested that *K. pneumoniae* has a high degree of environmental stability under some circumstances and indicates that decontamination should be verified after the cleaning process (2).

The capsule polysaccharide of *K. pneumoniae* has long been viewed as an important virulence factor that promotes resistance to phagocytosis and serum bactericidal activity. To date, more than 100 capsular (K) serotypes of *K. pneumoniae* have been identified (18). Notably, many reports have shown that K1 and K2 serotypes are strongly associated with hvKP (19, 20). Nevertheless, serotype K57 has also been considered as one of the pyogenic liver abscess-associated capsular types that are rarely reported (21, 22). In 2020, Wei et al. studied 39 K57 *K. pneumoniae* isolates. Among them, ST412 was the most prevalent, and all isolates harbored *bla*_KPC−2_ (22). In the present study, we identified that all 32 CR-hvKP isolates belonged to K57 and ST11. To our knowledge, this is the first report of K57 and ST11 *K. pneumoniae* with carbapenem resistance associated with clinical infection.

Several virulence factors have been suggested to contribute to the pathogenesis of hvKP strains. To date, *rmpA* and *magA* are the most frequently reported factors that have direct correlation with hvKP virulence (23). *RmpA* is located either on the chromosome or on a large virulence plasmid. The correlation between *rmpA* and hypermucoviscosity is very high, and the presence of *rmpA* among a set of genes has been proposed as biomarker to identify potential hvKp strains (24). However, in this study, all 32 hypermucoviscosity-negative isolates were positive for *rmpA* and a previous study reported similar results (22). The underlying reason may be that *rmpA* cannot work alone, or there may be a mutation of *rmpA*. In addition, a correlation between the presence of *rmpA* and aerobactin has been described, wherein 96% of *rmpA* positive isolates coproduced aerobactin (13). In this study, all 32 isolates were aerobactin producers.

In addition, the 32 isolates expressed four other virulence genes, including *fimH, mrkD, uge*, and *wabG*. *FimH* and *mrkD* encode type I and type III fimbriae, respectively. Fimbriae allow bacteria to attach to the host cells to establish infection. A previous report confirmed observations that type III fimbriae contribute to biofilm formation, and demonstrated that the expression of type III fimbriae is positively correlated with iron concentration (7). In this study, capsule-associated genes *uge* and *wabG* were detected in 32 isolates simultaneously. A previous study showed that the deletion of genes for capsule synthesis decreased the virulence of *K. pneumoniae* to varying degrees (25). In the absence of the gene *uge, K. pneumoniae* is less capable of causing urinary tract infections, pneumonia, and sepsis (26). In general, the serotype of the 32 isolates was K57, and their virulence genes were the same. However, the virulence test performed by infecting *G. mellonella* larvae showed that the virulence of 11 isolates from six patients was different, and their virulence was closely related to the survival time of the patients.

All CR-hvKP isolates in the present study were *bla*_KPC−2_-producing ST11 *K. pneumoniae*. In addition to *bla*_KPC−2_, all 32 isolates harbored *bla*_SHV−11_ and *bla*_TEM−1_. This is similar to previous reports (8, 10). These resistance genes lead to the resistance to β-lactams in the isolates. It should be noted that the drug resistance genes carried by several isolates from the environment are slightly different from isolates from patients. For example, Kp15, Kp16 and Kp17 isolated from the environment of patient 3 carry two ESBL genes at the same time. Therefore, it is considered that CRKp may lose or acquire drug resistance genes in the process of transmission. Furthermore, all isolates were resistant to aminoglycosides and quinolones, which severely limited the clinical choice of antibiotics. Fortunately, all strains were sensitive to tigecycline, colistin and ceftazidime/averbatan. Finally, two patients recovered and was discharged, three patients died, and the mortality rate was 50.0%. This is higher than that described in previous reports (5, 26).

## Conclusions

We described an outbreak of ST11 *K. pneumoniae*, which exhibited hypervirulence and carbapenem resistance, in EICU of a teaching hospital in China. The serotype of all 32 isolates was K57. This report confirmed that CR-hvKP can be transmitted nosocomially through the surrounding environment of the hospital. Hence, monitoring the nosocomial dissemination of CR-hvKP is essential for its prevention and outbreak management.

## Data Availability Statement

The original contributions presented in the study are included in the article/Supplementary Material, further inquiries can be directed to the corresponding author/s.

## Ethics Statement

The studies involving human participants were reviewed and approved by the Ethics Committee of Taian City Central Hospital. The patients/participants provided their written informed consent to participate in this study. The animal study was reviewed and approved by the Ethics Committee of Taian City Central Hospital.

## Author Contributions

CS carried out the experiments and wrote the paper. YJ performed the results analysis. WW collected clinical information of patients. MJ and SZ designed the experiments and revised the manuscript. All authors contributed to the article and approved the submitted version.

## Conflict of Interest

The authors declare that the research was conducted in the absence of any commercial or financial relationships that could be construed as a potential conflict of interest.

## Publisher's Note

All claims expressed in this article are solely those of the authors and do not necessarily represent those of their affiliated organizations, or those of the publisher, the editors and the reviewers. Any product that may be evaluated in this article, or claim that may be made by its manufacturer, is not guaranteed or endorsed by the publisher.

## Abbreviations

cKP: classical *K. pneumoniae*
CRKp: carbapenem-resistant *K. pneumoniae*
hvKP: hypervirulent *K. pneumoniae*
CR-hvKP: carbapenem-resistant hypervirulent *K. pneumoniae*
EICU: emergency intensive care unit
BALF: bronchoalveolar lavage fluid
MLST: multilocus sequence typing
PCR: polymerase chain reaction
PFGE: pulse-field gel electrophoresis.
