# Supplementary material for: An Outbreak of Carbapenem-Resistant Klebsiella pneumoniae of K57 Capsular Serotype in an Emergency Intensive Care Unit of a Teaching Hospital in China
Source: Front Public Health. 2021 Sep 1;9:724212. doi: 10.3389/fpubh.2021.724212 (PMC8441004; doi:10.3389/fpubh.2021.724212)
Supplement: Supplementary file 1 [file Table_1.DOC]

**Supplementary Table 1 The antibiotic susceptibility of 32 CR-hvKP isolates (μg/ml).**

| isolate No. | CZO | CXM | CRO | CAZ | FEP | FOX | ATM | SAM | TZP | ETP | IPM | MEM | CEN | AMK | CIP | LEV | SXT | TGC | COL | CZA* |
| --- | --- | --- | --- | --- | --- | --- | --- | --- | --- | --- | --- | --- | --- | --- | --- | --- | --- | --- | --- | --- |
| Kp1 | >64 | >16 | >32 | >16 | >64 | >16 | >16 | >16/8 | >64 | >32 | >32 | >32 | >8 | >32 | >2 | >4 | <=2/38 | 0.25 | 0.5 | 23 |
| Kp2 | >64 | >16 | >32 | >16 | >64 | >16 | >16 | >16/8 | >64 | >32 | >32 | >32 | >8 | >32 | >2 | >4 | <=2/38 | 0.5 | 0.5 | 24 |
| Kp3 | >64 | >16 | >32 | >16 | >64 | >16 | >16 | >16/8 | >64 | >32 | >32 | >32 | >8 | >32 | >2 | >4 | <=2/38 | 0.5 | 0.5 | 24 |
| Kp4 | >64 | >16 | >32 | >16 | >64 | >16 | >16 | >16/8 | >64 | >32 | >32 | >32 | >8 | >32 | >2 | >4 | <=2/38 | 0.25 | 0.5 | 23 |
| Kp5 | >64 | >16 | >32 | >16 | >64 | >16 | >16 | >16/8 | >64 | >32 | >32 | >32 | >8 | >32 | >2 | >4 | >2/38 | 0.5 | 0.5 | 23 |
| Kp6 | >64 | >16 | >32 | >16 | >64 | >16 | >16 | >16/8 | >64 | >32 | >32 | >32 | >8 | >32 | >2 | >4 | <=2/38 | 0.25 | 1 | 24 |
| Kp7 | >64 | >16 | >32 | >16 | >64 | >16 | >16 | >16/8 | >64 | >32 | >32 | >32 | >8 | >32 | >2 | >4 | <=2/38 | 0.5 | 0.5 | 25 |
| Kp8 | >64 | >16 | >32 | >16 | >64 | >16 | >16 | >16/8 | >64 | >32 | >32 | >32 | >8 | >32 | >2 | >4 | <=2/38 | 0.5 | 0.5 | 26 |
| Kp9 | >64 | >16 | >32 | >16 | >64 | >16 | >16 | >16/8 | >64 | >32 | >32 | >32 | >8 | >32 | >2 | >4 | <=2/38 | 0.5 | 1 | 25 |
| Kp10 | >64 | >16 | >32 | >16 | >64 | >16 | >16 | >16/8 | >64 | >32 | >32 | >32 | >8 | >32 | >2 | >4 | <=2/38 | 1 | 0.5 | 26 |
| Kp11 | >64 | >16 | >32 | >16 | >64 | >16 | >16 | >16/8 | >64 | >32 | >32 | >32 | >8 | >32 | >2 | >4 | <=2/38 | 1 | 0.5 | 26 |
| Kp12 | >64 | >16 | >32 | >16 | >64 | >16 | >16 | >16/8 | >64 | >32 | >32 | >32 | >8 | >32 | >2 | >4 | <=2/38 | 1 | 0.5 | 23 |
| Kp13 | >64 | >16 | >32 | >16 | >64 | >16 | >16 | >16/8 | >64 | >32 | >32 | >32 | >8 | >32 | >2 | >4 | <=2/38 | 0.5 | 0.5 | 24 |
| Kp14 | >64 | >16 | >32 | >16 | >64 | >16 | >16 | >16/8 | >64 | >32 | >32 | >32 | >8 | >32 | >2 | >4 | <=2/38 | 0.5 | 0.5 | 23 |
| Kp15 | >64 | >16 | >32 | >16 | >64 | >16 | >16 | >16/8 | >64 | >32 | >32 | >32 | >8 | >32 | >2 | >4 | <=2/38 | 0.25 | 1 | 24 |
| Kp16 | >64 | >16 | >32 | >16 | >64 | >16 | >16 | >16/8 | >64 | >32 | >32 | >32 | >8 | >32 | >2 | >4 | <=2/38 | 0.5 | 0.5 | 26 |
| Kp17 | >64 | >16 | >32 | >16 | >64 | >16 | >16 | >16/8 | >64 | >32 | >32 | >32 | >8 | >32 | >2 | >4 | <=2/38 | 0.5 | 0.5 | 24 |
| Kp18 | >64 | >16 | >32 | >16 | >64 | >16 | >16 | >16/8 | >64 | >32 | >32 | >32 | >8 | >32 | >2 | >4 | <=2/38 | 0.25 | 0.5 | 24 |
| Kp19 | >64 | >16 | >32 | >16 | >64 | >16 | >16 | >16/8 | >64 | >32 | >32 | >32 | >8 | >32 | >2 | >4 | <=2/38 | 0.5 | 0.5 | 25 |
| Kp20 | >64 | >16 | >32 | >16 | >64 | >16 | >16 | >16/8 | >64 | >32 | >32 | >32 | >8 | >32 | >2 | >4 | <=2/38 | 0.25 | 0.5 | 23 |
| Kp21 | >64 | >16 | >32 | >16 | >64 | >16 | >16 | >16/8 | >64 | >32 | >32 | >32 | >8 | >32 | >2 | >4 | >2/38 | 0.5 | 1 | 23 |
| Kp22 | >64 | >16 | >32 | >16 | >64 | >16 | >16 | >16/8 | >64 | >32 | >32 | >32 | >8 | >32 | >2 | >4 | >2/38 | 0.5 | 0.5 | 25 |
| Kp23 | >64 | >16 | >32 | >16 | >64 | >16 | >16 | >16/8 | >64 | >32 | >32 | >32 | >8 | >32 | >2 | >4 | >2/38 | 0.5 | 0.5 | 26 |
| Kp24 | >64 | >16 | >32 | >16 | >64 | >16 | >16 | >16/8 | >64 | >32 | >32 | >32 | >8 | >32 | >2 | >4 | <=2/38 | 1 | 0.5 | 23 |
| Kp25 | >64 | >16 | >32 | >16 | >64 | >16 | >16 | >16/8 | >64 | >32 | >32 | >32 | >8 | >32 | >2 | >4 | >2/38 | 1 | 0.5 | 24 |
| Kp26 | >64 | >16 | >32 | >16 | >64 | >16 | >16 | >16/8 | >64 | >32 | >32 | >32 | >8 | >32 | >2 | >4 | <=2/38 | 1 | 0.5 | 24 |
| Kp27 | >64 | >16 | >32 | >16 | >64 | >16 | >16 | >16/8 | >64 | >32 | >32 | >32 | >8 | >32 | >2 | >4 | <=2/38 | 0.5 | 0.5 | 25 |
| Kp28 | >64 | >16 | >32 | >16 | >64 | >16 | >16 | >16/8 | >64 | >32 | >32 | >32 | >8 | >32 | >2 | >4 | <=2/38 | 0.5 | 1 | 25 |
| Kp29 | >64 | >16 | >32 | >16 | >64 | >16 | >16 | >16/8 | >64 | >32 | >32 | >32 | >8 | >32 | >2 | >4 | >2/38 | 0.25 | 0.5 | 25 |
| Kp30 | >64 | >16 | >32 | >16 | >64 | >16 | >16 | >16/8 | >64 | >32 | >32 | >32 | >8 | >32 | >2 | >4 | >2/38 | 0.5 | 0.5 | 26 |
| Kp31 | >64 | >16 | >32 | >16 | >64 | >16 | >16 | >16/8 | >64 | >32 | >32 | >32 | >8 | >32 | >2 | >4 | <=2/38 | 0.5 | 0.5 | 23 |
| Kp32 | >64 | >16 | >32 | >16 | >64 | >16 | >16 | >16/8 | >64 | >32 | >32 | >32 | >8 | >32 | >2 | >4 | <=2/38 | 0.25 | 0.5 | 23 |

MIC:Minimum inhibitory concentration; CZO:cefazolin; CXM:cefuroxime; CRO:ceftriaxone; CAZ:ceftazidime; FEP:cefepime; FOX:cefoxitin; ATM:aztreonam; SAM:ampicillin sulbactam; TZP:piperacillin/tazobactam; ETP:ertapenem; IPM:imipenem; MEM:meropenem; CEN:gentamicin; AMK:amikacin; CIP:ciprofloxacin; LEV:levoﬂoxacin; SXT: trimethoprim-sulfamethoxazole; TGC: tigecycline; COL: colisin; CZA: ceftazidime/avibatan. *：diameter of bacteriostatic zone.
